# A High Density Genetic Map Derived from RAD Sequencing and Its Application in QTL Analysis of Yield-Related Traits in *Vigna unguiculata*

**DOI:** 10.3389/fpls.2017.01544

**Published:** 2017-09-07

**Authors:** Lei Pan, Nian Wang, Zhihua Wu, Rui Guo, Xiaolu Yu, Yu Zheng, Qiuju Xia, Songtao Gui, Chanyou Chen

**Affiliations:** ^1^Hubei Province Engineering Research Centre of Legume Plants, College of Life Sciences, Jianghan University Wuhan, China; ^2^Department of Forestry, College of Horticulture and Forest, Huazhong Agriculture University Wuhan, China; ^3^National Key Laboratory of Crop Genetic Improvement, Center of Integrative Biology, College of Life Science and Technology, Huazhong Agricultural University Wuhan, China; ^4^Institute for Interdisciplinary Research, Jianghan University Wuhan, China; ^5^BGI-Agro Shenzhen, China; ^6^Department of Genetics, State Key Laboratory of Hybrid Rice, College of Life Sciences, Wuhan University Wuhan, China

**Keywords:** *Vigna unguiculata*, RAD sequencing, single nucleotide polymorphisms, linkage mapping, pod yield-related traits, quantitative trait loci

## Abstract

Cowpea [*Vigna unguiculata* (L.) Walp.] is an annual legume of economic importance and widely grown in the semi-arid tropics. However, high-density genetic maps of cowpea are still lacking. Here, we identified 34,868 SNPs (single nucleotide polymorphisms) that were distributed in the cowpea genome based on the RAD sequencing (restriction-site associated DNA sequencing) technique using a population of 170 individuals (two cowpea parents and 168 F_2:3_ progenies). Of these, 17,996 reliable SNPs were allotted to 11 consensus linkage groups (LGs). The length of the genetic map was 1,194.25 cM in total with a mean distance of 0.066 cM/SNP marker locus. Using this map and the F_2:3_ population, combined with the CIM (composite interval mapping) method, eleven quantitative trait loci (QTL) of yield-related trait were detected on seven LGs (LG4, 5, 6, 7, 9, 10, and 11) in cowpea. These QTL explained 0.05–17.32% of the total phenotypic variation. Among these, four QTL were for pod length, four QTL for thousand-grain weight (TGW), two QTL for grain number per pod, and one QTL for carpopodium length. Our results will provide a foundation for understanding genes related to grain yield in the cowpea and genus *Vigna*.

## Introduction

Cowpea [*Vigna unguiculata* (L.) Walp. 2X = 2N = 22], ~587 Mb in genome size, is an important drought-tolerant legume crop (Iwata et al., 2013). It is one of the top five food legumes or pulses grown worldwide (Smýkal et al., 2015). Cowpea is cultivated mainly for fresh and dry grains, leaves, and fodder (Lucas et al., 2011). Due to its symbiotic nitrogen fixation, the crop is a valuable component of rotations and intercrops with sorghum and millets. The nitrogen fixation level of cowpea is less than soybean, but more than common bean (Quaye et al., 2009; Iwata et al., 2013). Cowpea is widely grown throughout tropical and subtropical regions, including Africa and some Asian, South European, Central and South American countries (Tosti and Negri, 2005). In many regions, especially in Africa and Asia, cowpea plays an important role in providing protein-rich components against protein-poor diet (Ehlers and Hall, 1997). The West African sub-region contributes to predominantly global cowpea yield (~95%), and Nigeria has the maximum mass of cowpea product around the world (FAOSTAT, 2012). Two cowpea subspecies are generally cultivated around the world. One is a grain-type cowpea that is harvested for matured seeds and is commonly known as the African cowpea or the common cowpea (*V. unguiculata* L. Walp. ssp. *unguiculata*) (Singh, 2002). The other is a vegetable-type cowpea known as the asparagus bean or “yardlong” bean (*V. unguiculata* L. Walp. ssp. *sesquipedalis*), which is planted for its long tender immature pods (Xu et al., 2016).

Currently, one of the major goals in cowpea breeding is to improve cowpea yield by using modern genetic strategies, such as marker-assisted selection and genomic-assisted selection. Selection of genomic loci or genes related to the traits of interest for economic importance requires using genetic maps constructed with molecular markers (Chapman et al., 2008). Therefore, genetic maps play a basic role in the progress of crop molecular breeding (Andriantahina et al., 2013).

In the past two decades, with progress in marker technology, genetic mapping in cowpea has resulted in increasingly dense linkage maps (Lucas et al., 2011). It is noteworthy that several different types of DNA molecular markers have been applied for different mapping populations (F_2_ population, BC_1_F_1_ population, and recombination inbred line) in cowpea (Ouédraogo et al., 2002; Muchero et al., 2009a; Xu et al., 2011; Kongjaimun et al., 2012a).

The quantitative trait loci (QTL) related to growth and abiotic/biotic stress tolerance have been identified in cowpea. Twelve QTL controlling resistance to drought have been identified (Muchero et al., 2009b), whereas only five QTL (*Cht–1*~*Cht–5*) for resistance to high temperature were detected (Lucas et al., 2013a). Nine QTL (*Mac*-1~*Mac*-9) for *Macrophomina phaseolina* resistance (Muchero et al., 2011) and three QTL (*Fot* 3-1, *Fot* 4-1, and *Fot* 4-2) for *Fusarium oxysporum* resistance (Pottorff et al., 2012a, 2014a) were discovered. QTL for development-related traits have been focused on cowpea in recent years, including pod length (Kongjaimun et al., 2012a), leaf morphology (Pottorff et al., 2012b), seed size (Kongjaimun et al., 2012b), horticultural traits (Xu et al., 2013), pod tenderness, total soluble solid (Kongjaimun et al., 2013), flowering time (Andargie et al., 2013), and seed coats (Pottorff et al., 2014b). Although previous genetic maps have been successfully applied to identify QTL for abiotic/biotic resistance and growth traits in cowpea, the available markers of these maps, restricted from hundreds to a few thousand, make it difficult to locate the genomic regions tightly linked to crucial traits. Thus, the development of high resolution genetic maps is of utmost importance for cowpea breeding programs.

The advancement of next-generation sequencing (NGS) technologies has accelerated the identification of SNPs and genotyping process (Peterson et al., 2012). The combination of high-throughput NGS technologies and restriction enzyme digestion is able to reduce the complexity of the target genomes, and to develop a large number of SNP markers in any species of interest (Hyten et al., 2010; Davey et al., 2011; Chen et al., 2013; Raman et al., 2014). Restriction-site associated DNA sequencing (RAD-seq) is one of the preferred NGS technologies for high-throughput genotyping. The RAD-seq method, first described by Baird et al. (2008), has been used in many species without a reference genome, including rygrass (Pfender et al., 2011), barley (Chutimanitsakun et al., 2011), eggplant (Barchi et al., 2012), grape (Wang N. et al., 2012), and sesame (Wu et al., 2014). It has been a useful tool for linkage map construction, QTL mapping and comparative genomics (Gonen et al., 2014).

To date, despite the available genetic map based on SNPs in cowpea (Muchero et al., 2009a; Lucas et al., 2011), the large-scale discovery, and utilization of SNP markers have not been carried out to build a fine linkage map of cowpea. Consequently, in the present study, we aimed to construct a high-density SNP linkage map of the cowpea genome using RAD-seq analysis with two parents a population of 168 F_2:3_ lines. Then, in a further analysis, we identified yield-related QTLs based on the genomic resources of cowpea.

## Materials and methods

### Plant samples and field trials

**Figure 1:**
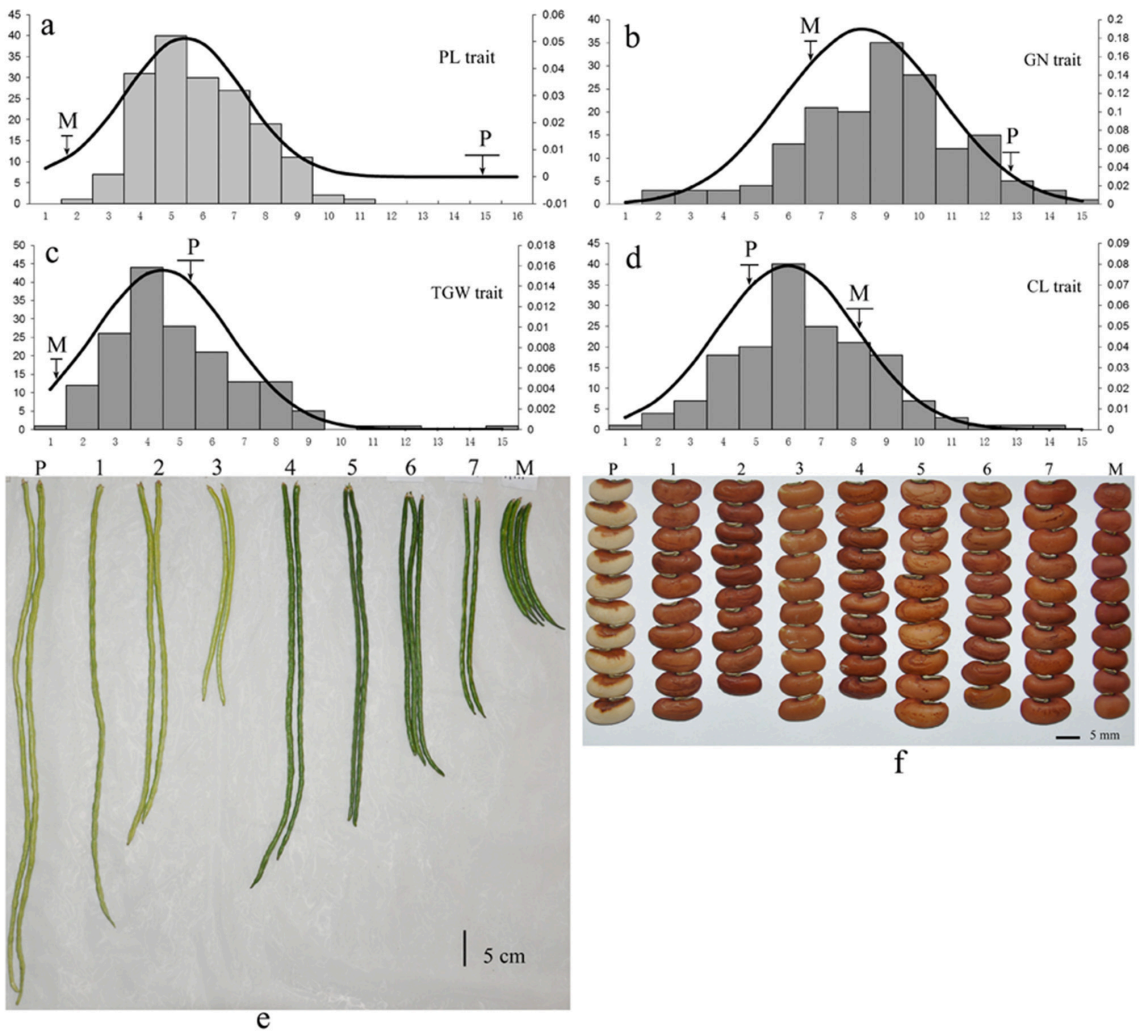
Phenotypic variation of yield-related traits in cowpea. The lower-case letters “**a–f**” represent the phenotypic variation of the four traits (PL, GN, TGW, and CL) among the 168 F_2:3_ offspring. P: Yardlong bean (*V. unguiculata* ssp. *sesquipedalis*) cultivar “Xiabao II” (male). M: “Green pod cowpea” (female). The codes 1~7 represent seven F_2:3_ offspring derived from the cross between “Green pod cowpea” (female) and cultivar “Xiabao II” (male).

The mapping population consisted of 168 F_2:3_ progenies from a cross between two cowpea subspecies accessions: (*V. unguiculata* ssp. *unguiculata*) cultivar “Green pod cowpea” (female) and yardlong bean (*V. unguiculata* ssp. *sesquipedalis*) cultivar “Xiabao II” (male) (Figure 1, Supplementary Figure 1). The two parents showed distinct morphological traits, including seed coat color, pod length, carpopodium length, grain number per pod and grain size.

Field trials were performed in the Caidian district (114.03E, 30.58N), Wuhan city, Hubei province, P.R. China from July to October 2014. Each sample was planted 1 m apart in rows 1.5 m in length.

### Ethics statement

The authors declare that the experiments comply with the current laws of the country in which they were performed.

### Trait evaluation

For each F_2:3_ progeny and the two parents, trait evaluation included pod length (PL, cm), carpopodium length (CL, cm), grain number per pod (GN), and thousand-grain weight (TGW, g). PL was measured as the average length from the top to the end of ten pods. CL was measured as the average length from the upper part to the end of carpopodium of ten pods. GN was measured as the average number of seeds of 10 uniform capsules from each plant. To measure the TGW, the mean weight of three independent samples of fifty grains was calculated first, and then was converted to TGW. All samples of F_2:3_ population were tested for phenotyping at the harvest stage.

### RAD library construction and sequencing

Young leaves were collected from each sample and then frozen at −80°C. Total genomic DNA was extracted from each sample using the modified CTAB method as described by Doyle and Doyle (1987). Genomic DNA from each sample was quantified using spectrophotometry (Qubit 2.0 Fluorometer, Invitrogen) and checked for genomic integrity by 0.8% agarose gel electrophoresis.

Restriction-site associated DNA (RAD) strategy combined with Illumina DNA sequencing was used for the fast and effective identification of SNP markers. RAD libraries were constructed following a protocol from Baird et al. (2008). The general protocol was as follows: first, each sample of genomic DNA (~0.5 μg per sample) from the 168 individuals and their two parents was digested with 20 units (U) of *EcoR*I or *Sbf* I (New England Biolabs, NEB) for 15 min at 37°C in a 50-μL (microlitre) reaction and then heat denatured at 65°C. P1 Adapters, each with a unique 4–8 bp (base pair) molecular-identifying sequence (MID), were ligated to the genomic DNA of each sample. The adapter sequences were as follows: *EcoR*I digestion, top: 5′-AATGATACGGCGACCACCGAGATCTACACTCTTTCCCTACACGACGCTCTTCCGATCTxxxxx-3′ [x = barcode], bottom: 5′-Phos-AATTxxxxxAGATCGGAAGAGCGTCGTGTAGGGAAAGAGTGTAGATCTCGGTGGTCGCCGTATCATT-3′, for *Sbf* I digestion, top: 5′-AATGATACGGCGACCACCGAGATCTACACTCTTTCCCTACACGACGCTCTTCCGATCTxxxxTGCA-3′, bottom: 5′-Phos-xxxxAGATCGGAAGAGCGTCGTGTAGGGAAAGAGTGTAGATCTCGGTGGTCGCCGTATCATT-3′. The ligation reaction was incubated at room temperature (RT) for 20 min in a final volume of 10 μL containing 2.5 μL P1Adapter (100 nM), 1 μL rATP (100 mM) (Promega), 1 μL 10 × *EcoR*I buffer, 0.5 μL T4 DNA Ligase (1,000 U) (NEB), and 5 μL H_2_O. Samples were treated with heat denaturation at 65°C for 20 min again. The pooled samples of each library were randomly cut to DNA fragments of 400–700 bp. The size of sheared DNA was isolated through 1% agarose gel electrophoresis using a DNA gel extraction kit (Tiangen, China). The ends of the DNA were blunted by using the Quick Blunting Kit (NEB). After purifying the samples using a DNA purification kit (Tiangen), 15 U of Klenow exo^−^ (NEB) was added for adenine (Fermentas) overhangs on the 3′end of the DNA at 37°C. Then, 1 μL P2 Adapter (10 nM) was ligated to the DNA fragments at RT. Samples were again purified and eluted in 50 μL. Five microlitres of this product was used in a PCR amplification along with 50 μL Phusion Master Mix (NEB), 40 μL H_2_O and 5 μL primer mix (10 μM) (P1-forward primer: 5′-AATGATACGGCGACCACCGA-3′; P2-reverse primer: 5′-CAAGCAGAAGACGGCATACGA-3′). The PCR enrichment was carried out following instructions (NEB) for 11 cycles in total. Samples were gel purified, excising DNA 400–700 bp, and diluted to 10 nM.

Each library of the RAD products from the 170 individuals was sequenced on an individual lane of the Illumina HiSeq2000 next-generation sequencing platform (BGI-Shenzhen, Shenzhen, P.R. China). Sequencing data for each individual were then obtained in terms of the specific MID. Raw sequences are available in the National Centre for Biotechnology Information (NCBI) Sequence Read Archive (SRA) database under the accession number SRR5097134.

### SNP discovery and genotyping

Illumina raw sequence reads were filtered out at first if lacking expected restriction enzyme or ambiguous barcodes. Thus, the raw reads were ensured to more than 90% of the nucleotides having a quality value above Q30 (equals 0.1% sequencing error) and more than 95% above Q20 (equals 1% sequencing error). SNPs were discovered using a two-step process. In step 1, four individuals having the maximum sequencing reads were used to generate “reference tags” using Ustacks and Cstacks (Catchen et al., 2011). Then, the SNPs of the two parents and their offspring were identified by aligning the clean reads of the offspring to the reference tags. SNPs were regarded as true polymorphisms when each allele was represented by a minimum coverage of three reads or alleles were observed in at least five reads with a score >20 (*p* > 0.05). Genotypes of offspring individuals were determined by comparing to the parental genotypes. SNP markers with <10% missing data among the 170 individuals and meeting the above criteria were used in the following linkage mapping.

### Linkage mapping

The construction of genetic linkage map was performed by using JoinMap4 (Van Ooijen, 2006) and MSTmap (Wu et al., 2008). Linkage groups (LGs) were first defined using a minimum LOD threshold of 15 and a maximum recombination of 45% with the software JoinMap4. The Kosambi mapping function in the software MSTmap was used to convert recombination frequencies into map distance for each linkage group. The final marker order of each linkage group was verified by the software program RECORD (Van et al., 2005). The final marker order was verified in each linkage group by using RECORD (Van et al., 2005). The linkage map was graphically visualized with MapChart2.2 (Voorrips, 2002). The corrected length of the linkage map was assessed by multiplying the length of each linkage group by (*m* + 1)/(*m*−1), where *m* is the number of markers in the linkage group (Chakravarti et al., 1991). The coverage of the genome by the linkage map was then estimated by calculating *c* = 1 −*e*^−2*dn*/*L*^, where *d* is the average interval of markers, *n* is the number of markers, and *L* is the length of the linkage map (McDaniel et al., 2007).

To verify the map quality, we compared the new SNP map and recently reported cowpea maps (Muñoz-Amatriaín et al., 2017). We identified identical and proximal markers between the linkage maps using a reference genome of a related species as an intermediary (e.g., common bean) because the cowpea genome shares a high degree of collinearity with the common bean (Vasconcelos et al., 2015).

To assess the quality and universality of genetic maps, comparison of marker names, sequences, LGs and cM positions were carried out between our RAD-based SNP map and a recent published genetic map of cowpea (Muñoz-Amatriaín et al., 2017). Comparisons of linkage group composition and order were investigated for *P. vulgaris*. The basic workflow was as follows. First, all marker sequences were combined into a single FASTA file and mapped to a reference genome (e.g., common bean genome, NCBI accession number: ANNZ00000000.1) and published scaffolds (https://www.ncbi.nlm.nih.gov/genome/genomes/380). When two markers mapped to the same reference genome scaffold or contig, the two closest markers were considered as a marker pair. Each marker can only be paired once, and any other marker that was seconds closest (or further) to the now-paired marker is discarded. The paired markers were visualized by MapChart (Voorrips, 2002).

### QTL analysis

The mean phenotypic data from all the 170 individuals (two parents and 168 F_2:3_ progenies) were calculated for frequency distributions using function formula “NORMDIST” in Excel 2010.

Quantitative trait loci (QTL) were detected for each of the traits using the CIM (composite interval mapping) method implemented in Windows QTL Cartographer 2.5 (Wang S. et al., 2012). The LOD significance thresholds (*P* < 0.05) were analyzed by running 1,000 permutation tests. QTL regions were visualized by the program Matplotlib (version 1.4.3) in software Python version 2.7.10 (Sanner, 1999).

### Comparative analysis

To compare the genome structures of cowpea and a closely related species, a comparative analysis was carried out between the cowpea linkage map and the whole genome sequences of adzuki bean *V. angularis*. BLASTN was used to perform similarity searches (using the nucleotide sequences from which the SNP markers were mapped in the current linkage map of cowpea) against each pseudomolecule of the genome sequences of *V. angularis*, with a threshold *e*-value of 1e-20. The graphical comparative maps were drawn using the Circos program (Ubi et al., 2000).

In addition, sequences of cowpea RAD tags located in QTL regions were chosen to reveal potential genetic information by using BLASTN. These sequences of the mapped markers of cowpea RAD-tags were searched against genomes of legumes deposited in the NCBI database using BLASTN with an *e*-value cut-off of 1e-10 (www.ncbi.nlm.nih.gov/blast/).

## Results

### RAD sequencing and genotyping

A total of 170 RAD-seq libraries from two parents and 168 offspring were constructed and sequenced on an Illumina HiSeq2000 platform. After data filtering, 768,592,488 raw reads, consisting of ~69.315 Gb of sequencing data, were individually assigned to RAD tags.

A total of 383,077 RAD-tags were obtained. Using all the tags as reference sequences, SNP loci derived from paired end reads of 170 individuals were identified. Overall, 34, 868 polymorphic SNPs between the offspring genotypes and the two parent genotypes were identified on basis of strict SNP selection and criteria.

### Linkage map construction and SNP analysis

The set of 34,868 SNPs were used for genetic map construction. A total of 17,996 SNPs were successfully mapped onto 11 different LGs, covering 1,194.25 cM of the cowpea genome with an average distance of 0.066 cM between adjacent markers (Table 1 and Supplementary Figure 2). The length of the individual LGs ranged between 76.19 cM (LG11) and 198.85 cM (LG1), with an average inter-locus distance of 0.03 (LG2) to 0.13 cM (LG10 and LG11). LG1 was the densest, having 3,343 SNP loci, with an average density of 0.42 cM, whereas LG11 had the least number of SNP loci (591). On average, each LG consisted of 1,636 SNP loci spanning 108.57 cM. The corrected length of the linkage map was estimated at 1,195.97 cM, which was converted to genome coverage of 86.3%. Loci names and genetic distance of SNP positions on the 11 LGs of the genetic map are listed in Supplementary File 1.

**Table 1:** Distribution of mapped SNP markers on the 11 linkage group of cowpea.

**Linkage group**	**No. of SNPs**	**Distance (cM)**	The corrected map distance (cM)^*^	**Average inter-loci distance**
LG1	3,343	198.85	198.97	0.059
LG2	3,264	113.79	113.86	0.035
LG3	2,181	132.35	132.48	0.061
LG4	2,095	162.33	162.49	0.077
LG5	1,374	84.85	84.97	0.062
LG6	1,329	103.60	103.75	0.078
LG7	1,099	84.35	84.51	0.077
LG8	1,081	76.00	76.14	0.070
LG9	952	71.63	71.78	0.075
LG10	687	90.31	90.58	0.131
LG11	591	76.19	76.45	0.131
Total	17,996	1194.25	1195.97	
Average	1,636	108.57	108.72	0.066

*LG, Linkage group*.

* *Chakravarti et al. (1991)*.

To reveal the characterization of SNPs in cowpea, we investigated distribution and percentages of different SNP types. We found that the 17,996 SNP markers were genome-wide distributed across the 11 LGs, implying 1,636 SNPs per linkage group (Table 1). The maximum number of SNPs (3,343) was found in LG1, while the minimum number of SNPs (591) was found in LG11. Among the SNPs, the dominant type was transition, and the C/T and G/A types contributed to 34.8 and 35.8% of the SNPs, respectively. The other four SNP types were transversion including C/G, G/T, C/A, and A/T. Their proportion varied from 6.2 to 7.8% accounting for 29.4% of all SNPs (Supplementary Table 1). The SNP-flanking sequences and the polymorphic sites are summarized in Supplementary File 2.

### Consensus genetic linkage map

**Figure 2:**
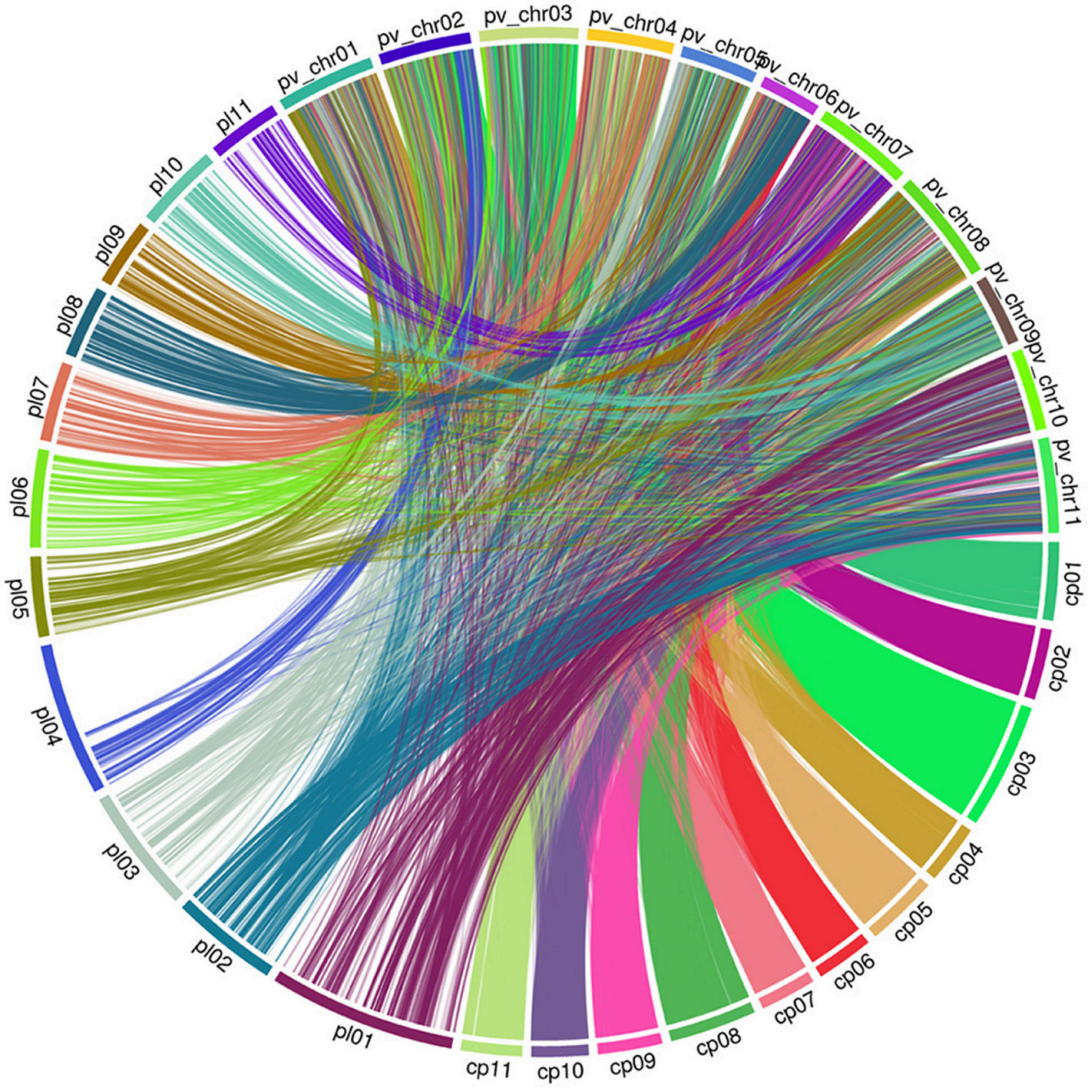
Genome view of synteny between two cowpea linkage groups and the common bean genome pl: Code of our linkage maps of cowpea. cp: Code of the consensus cowpea linkage maps (Muñoz-Amatriaín et al., 2017). pv_chr: Code of the Chromosomes of *Phaseolus vulgaris*.

To compare the maker position of our linkage map and the newly published consensus cowpea linkage maps (Muñoz-Amatriaín et al., 2017), we employed the partial reference genome available of *Phaseolus vulgaris* (Schmutz et al., 2014). To assign LGs to chromosomes, both our linkage map and the newly published consensus cowpea linkage maps (Muñoz-Amatriaín et al., 2017) were compared by mapping marker sequences against the common bean genome. Markers from both cowpea linkage maps were identified and aligned if they hit against the same contig/scaffold. Descriptive statistics for the identified markers on the LGs are in Table 2. Then, based on these markers, extensive synteny was observed using Circos (Krzywinski et al., 2009). A full comparison was completed across the two existing maps and the common bean genome. A set of 3,695 SNPs from our linkage map was identified, while 24,643 SNPs from the consensus cowpea linkage map were detected (Muñoz-Amatriaín et al., 2017). Thus, a total number of 28,338 SNPs were assigned to the 11 chromosomes of the common bean genome (Figure 2, Table 2, Supplementary File 3).

**Table 2:** Comparison of the current cowpea linkage map and the Muñoz-Amatriaín et al. (2017) map.

**Chromosome of *Phaseolus vulgaris***	**N1**	**N2**	**Total**
PvChr1	262	2,286	2,548
PvChr2	351	3,342	3,693
PvChr3	176	2,691	2,867
PvChr4	294	1,754	2,048
PvChr5	256	1,206	1,462
PvChr6	468	2,212	2,680
PvChr7	295	2,783	3,078
PvChr8	483	2,664	3,147
PvChr9	313	2,162	2,475
PvChr10	369	1,801	2,170
PvChr11	428	1,742	2,170
Total	3,695	24,643	28,338

*N1: Number of SNP markers from our linkage maps of cowpea matched with SNP markers on chromosomes of Phaseolus vulgaris*.

*N2: Number of SNP markers from the consensus cowpea linkage maps (Muñoz-Amatriaín et al., 2017) matched with SNP markers on chromosomes of Phaseolus vulgaris*.

*PvChr 1–11: Code of the Chromosomes of Phaseolus vulgaris*.

To capture identical markers between the two cowpea linkage maps, markers from each cowpea linkage map were paired if they hit against the same contig/scaffold of the common bean genome by taking the closest two markers together as each pair. We obtained 171 paired SNP marker loci. These paired markers were distributed unevenly across the common bean genome (Supplementary Figure 3, Supplementary File 4). This demonstrates that the current cowpea map accounts for 185 of the 37,372 SNPs mapped by Muñoz-Amatriaín et al. (2017) in cowpea, and the two maps are syntenic across all LGs to a degree.

### Identification of pod yield-related QTL using high-density genetic map in cowpea

All traits showed a continuous distribution and transgressive segregation in the F_2:3_ population (Figure 1) in this study, indicating that the traits were controlled by multiple genes. The near-normal curve distribution of PL, CL, GN, and TGW suggested a polygene mode for genetic control in these traits.

**Figure 3:**
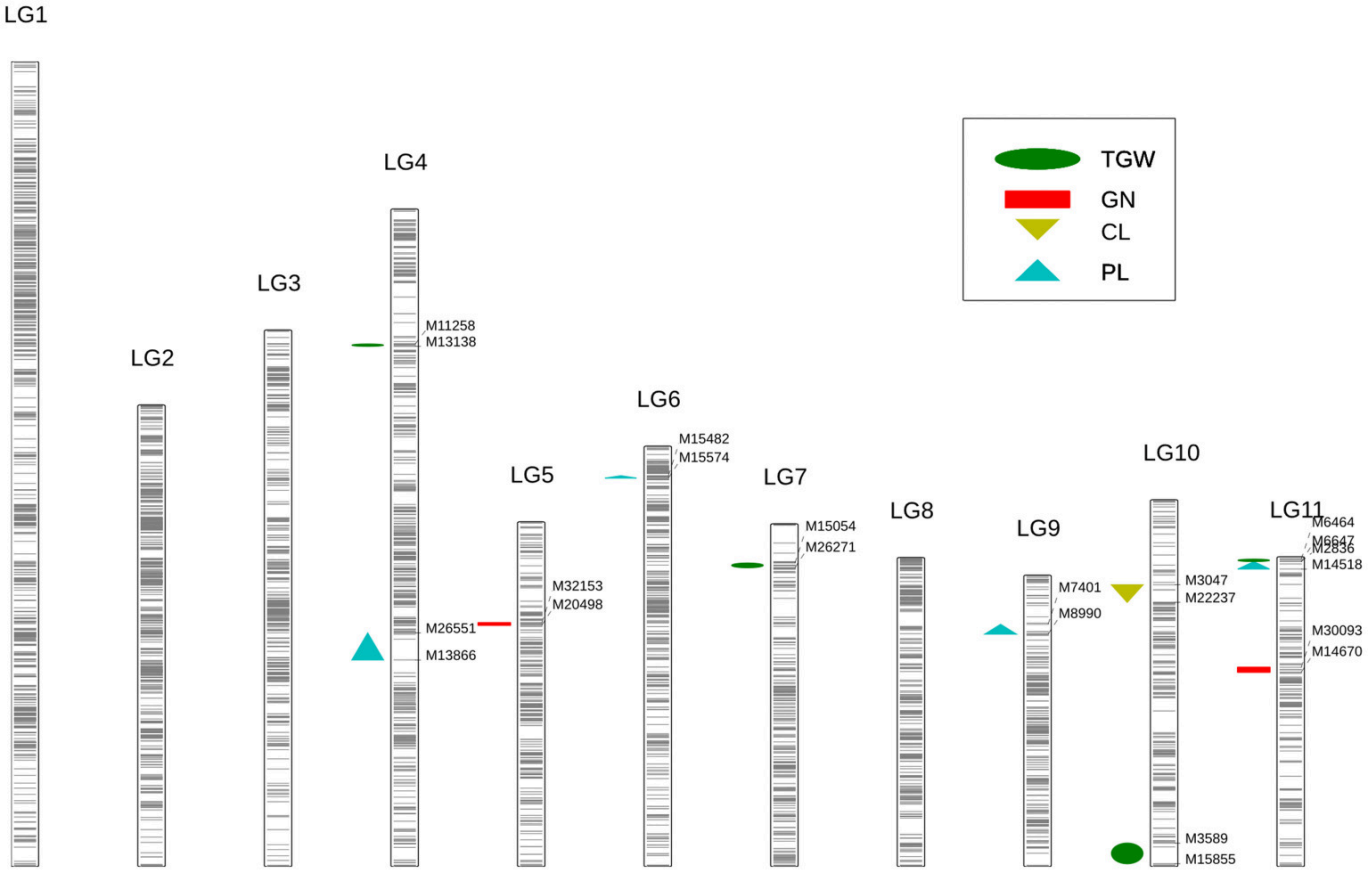
Identification of yield-related QTL using high-density genetic map in cowpea. Each black line represents a SNP locus. The green ellipse represents the trait of thousand-grain weight (TGW); the red rectangle represents the trait of grain number per pod (GN); the yellow triangle represents the trait of carpopodium length (CL); the blue triangle represents the trait of pod length (PL).

Overall, 11 significant QTL for cowpea pod yield were detected on seven LGs (LG4, 5, 6, 7, 9, 10, and 11) using the CIM method (Figure 3, Table 3, and Supplementary Figure 4). Most of these QTL were assigned on their respective LGs. On LG11, three QTL (*Qcpl-4, Qcgn-2*, and *Qctgw-4*) were located between the regions of 48.5–75.6 cM. On LG4, two QTL (*Qcpl-1* and *Qctgw-1*) were found between the regions of 56.1–128.9 cM. On LG9, one QTL (*Qcpl-3*) was detected on LG9 at the position of 57.9 cM. Noticeably, *Qcpl-3* located at 57.9 cM had the highest LOD value of 18.36 and correspondingly had the highest contribution to phenotypic variation at 17.32%. On LG10, two QTL (*Qccl-1 and Qctgw-3*) were detected at positions 4.4–68.2 cM. The other three LGs, (LG 5, 6, and 7,) contained one QTL locus, including *Qcgn-1* on LG5 at 59.8 cM, *Qcpl-2* on LG6 at 96.3 cM, and *Qctgw-2* on LG7 at 77.4 cM. Most of the 11 QTL showed negative additive effects except *Qcpl-2* (1.70) and *Qccl-1* (0.08).

**Table 3:** QTL of pod yield-related traits detected by CIM form the analysis of the F_2:3_ population in cowpea (LOD > 3).

**Traits**	**QTL**	**^a^LG**	**Genetic position (cM)**	**Marker interval**	**LOD**	**^b^Additive effect**	**^#^R^2^ %**
PL (pod length)	*Qcpl-1*	LG4	56.1	M13866-M26551	10.16	−3.25	3.73
	*Qcpl-2*	LG6	96.3	M15574-M15482	3.43	1.70	6.14
	*Qcpl-3*	LG9	57.9	M8990-M7401	18.36	−4.78	17.32
	*Qcpl-4*	LG11	74.5	M14518-M2836	9.60	−3.98	15.45
CL (carpopodium length)	*Qccl-1*	LG10	68.2	M22237-M3047	3.10	0.08	2.06
GN (grain number per pod)	*Qcgn-1*	LG5	59.8	M20498-M32153	3.36	−0.40	0.05
	*Qcgn-2*	LG11	48.5	M14670-M30093	4.40	−0.93	6.37
TGW (thousand grain weight)	*Qctgw-1*	LG4	128.9	M13138-M11258	4.07	−12.00	8.93
	*Qctgw-2*	LG7	77.4	M26271-M15054	4.50	−7.35	0.82
	*Qctgw-3*	LG10	4.4	M15855-M3589	3.49	−4.17	7.77
	*Qctgw-4*	LG11	75.6	M6647-M6464	3.54	−11.19	9.85

a LG, Linkage group;

b Additive effect, positive and negative indicated addictive effect;

*#R^2^ %, percentage of explained phenotypic variation*.

From these 11 QTL, four QTL for cowpea pod length (*Qcpl-1*≈*Qcpl-4*) explaining 42.64% of the total phenotypic variation and two major loci (explaining > 15% of the total variation) were detected. Moreover, we detected two major-effect QTL from the above mentioned four QTL that had a phenotypic effect (*R*^2^) of more than 15%, including *Qcpl-3* (17.32%), and *Qcpl-4* (15.45%).

### Comparative analysis between the sequences of the mapped SNPs in cowpea and related species

**Figure 4:**
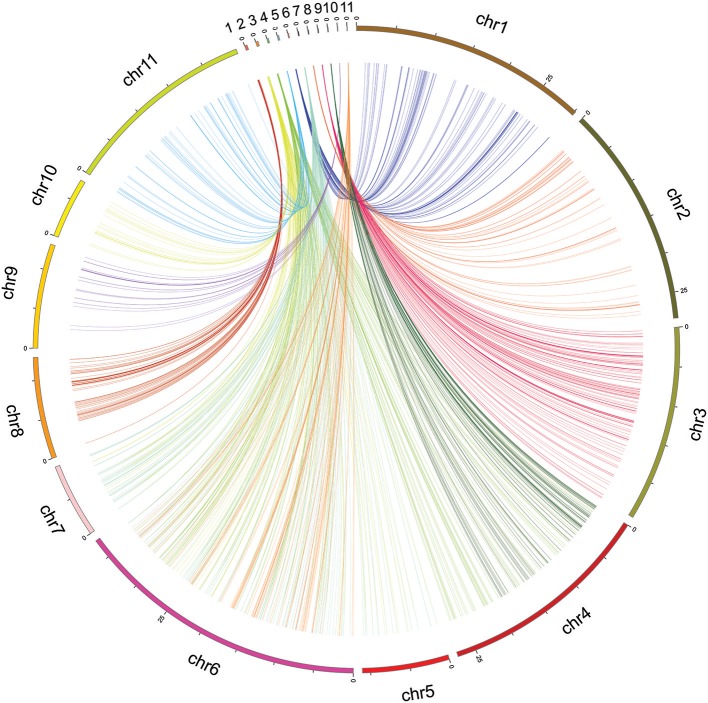
Circos mapping of eleven linkage groups of cowpea (top) to the adzuki bean genome.

The mapped SNP flanking sequences of *V. unguiculata* showed significant alignment with similar sequences in the adzuki bean (*Vigna angularis*) (Figure 4). Between *V. unguiculata* and *V. angularis*, 5,616 of 17,996 (31.2%) mapped SNP tags (94 bp per tag) were found, which had a total length of 527.9 Kb (Supplementary Table 2).

To uncover the synteny information between cowpea and the closely related species adzuki bean, all tags of the mapped SNPs of *V. unguiculata* were compared with the *V. angularis* genome. Based on the maximum number tags between the two species, all chromosomes of *V. angularis* were assigned to at least one linkage group of *V. unguiculata*, except chromosome 6 of *V. angularis*, which had *two* assigned LGs of cowpea (Supplementary Table 2), possibly due to translocation on the chromosome. Eleven LGs of *V. unguiculata* aligned to eleven chromosomes of *V. angularis* (Supplementary File 5), caused by two LGs (LG 2 and 6) sharing one single chromosome of *V. angularis* (chromosome 6). These conserved regions should represent homologous regions between the two genomes. Our result implies that, although both *V. unguiculata* and *V. angularis* contain the same number of chromosomes (2X = 2N = 22), the chromosomes of cowpea evolved independently and exhibit different rearrangements from the chromosomes of *V. angularis*.

In addition, 93 RAD-tags located in QTL regions of the four traits (PL, TGW, GN, and CL) were analyzed by BLASTN in the NCBI databases. Of these, eight RAD tags were found to have homology domains in two species (*V. radiata* and *V. angularis*) in genus *Vigna* (Supplementary File 6). These tags were mainly related to two traits (pod length and TGW). Noticeably, two RAD-tag (M26270 and M26271) sequences were revealed to show similarity with the sequences of E3 ubiquitin protein ligase DRIP2-like (LOC106770573), which targets transcription factors for degradation and thus regulates gene expression in response to environmental or hormonal signals (Qin et al., 2008).

## Discussion

### High-density SNP linkage map construction in cowpea based on RAD-seq

Molecular markers and genetic maps play a significant role in the genetic mapping of important traits in crops (Zhou et al., 2014). With recent significant advancements, RAD-seq technology as well as other technologies, such as GBS based DArTseq markers has been employed as a useful tool for high-density genetic mapping and 30 QTL mapping (Baird et al., 2008; Raman et al., 2014, 2016; Zou et al., 2016). This technology can discover thousands of markers in the model or other organisms regardless of genome size or reference genomes (Davey and Blaxter, 2010). Currently, the RAD-seq technique has been gradually applied to several legume species, such as chickpea (Gaur et al., 2012) and peanut (Gupta et al., 2015). However, to the best of our knowledge, no study has ever been reported on RAD-seq-based SNP discovery technique in cowpea. In the present study, we constructed and characterized a high-density SNP linkage map in cowpea using the RAD-seq method. A total of 383,077 RAD tags were obtained from the two cowpea parents and 168 F_2:3_ offspring individuals. Then, we identified 34,868 novel SNPs distributed throughout the whole cowpea genome, providing genetic variation resources for the genome-wide association investigations in the future. Of the discovered SNPs, 17,996 SNPs were successfully assigned to 11 LGs corresponding to 11 chromosome pairs of the cowpea genome. The map spanned 1194.25 cM, showing 0.066 cM/SNP locus. Therefore, the current version of the population-specific linkage maps for *V. unguiculata* was a considerable improvement compared with the formerly published linkage maps of cowpea (Table 4). The analysis of synteny between our linkage map and a recent map of cowpea (Muñoz-Amatriaín et al., 2017) revealed that the two maps show relatively high-level synteny across all cowpea LGs. This study generated a high density RAD-based linkage map of cowpea to date. The inter-marker distance of 0.066 cM indicated it would be beneficial for locating sequence scaffold on the physical map of the cowpea genome sequence (Deokar et al., 2014). Moreover, this high-density linkage map would be an ideal genetic map for QTL mapping, gene locating, and comparative genomics analysis for agriculturally important traits of cowpea. However, there are limitations for the utilization of this map because it was based on the SNPs between only two cowpea varieties, with a SNP flanking sequence of only 94 bp.

**Table 4:** Comparison of linkage maps for *Vigna unguiculata* from a single population.

**Cross combination**	**Population type (population size)**	**Type of markers (Number of markers)**	**Groups**	**Map length (cM)**	**References**
Two cowpea subspecies accessions	F_2:3_ (168)	SNP (17,996)	11	1,194.25 cM	the present study
IT84S-2049 × 524B	F_2_ (94)	RAPD (133), RFLP (36), AFLP (267)	11	2,670 cM	Ouédraogo et al., 2002
ZN016 × ZJ282	RIL (114)	SNP (191), SSR (184)	11	745 cM	Xu et al., 2013
13 RILs	RIL (1,293)	SNP (1,107)	11	680 cM	Lucas et al., 2011
JP81610 × TVnu457	BC_1_F_1_ (190)	SSR (226)	11	852.4 cM	Kongjaimun et al., 2012a
6 RILs	RIL (741)	SNP (928)	11	680 cM	Muchero et al., 2009a

*RIL, recombination inbred line*.

### Yield-related QTL based on high-density SNP genetic map in cowpea

The QTL related to domestication traits are non-randomness distribution across the crop genome (Gepts, 2004). This can be attributed to its association with “cultivation magnetism” and the “protracted transition paradigm” of crop domestication (Allaby, 2010). The increasing yield is one of the most significant domestication targets in crops, but it is a tedious process influenced by complex environmental components (Wu et al., 2014). Yield-related traits dominate QTL studies in wheat, maize, rice and soybeans, reflecting the agronomic and economic importance of these crops (Salvi and Tuberosa, 2015). Based on path coefficient analysis, it was revealed that single pod weight and number of pods per plant had the major positive contribution toward the pod yield in *Phaseolus vulgaris* (Ankit et al., 2017).

Quantitative trait loci (QTL) of yield-related traits have been detected in *Brassica napus* (Cai et al., 2016; Zhao et al., 2016), *Oryza nivara* (Ma et al., 2016), and *Hordeum vulgare* (Mikołajczak et al., 2016). In *B. napus*, 14 crucial candidate genes, which might be involved in developmental processes (such as, the regulation of flowering and vegetative phase change) and biomass accumulation (such as, response to secondsary wall biosynthesis), were revealed to be associated with the yield establishment (Lu et al., 2017). In several legume crops, agricultural traits have been investigated, including adzuki bean (Kaga et al., 2008), rice bean (Isemura et al., 2010), chickpea (Das et al., 2015), and cultivated peanut (Luo et al., 2017). In *Arachis hypogaea*, Luo et al. (2017) identified three co-localized major QTL for pod size and weight to a 3.7 cM interval on chromosome A05. These traits were controlled by a few large QTL or by a major QTL and several minor QTL. The QTL governing domestication traits in cowpea were reported in previous studies, which included pod length, plant height, seed size, flowering time, seed coat color, flower color and other traits (Ubi et al., 2000; Peksen, 2004; Kongjaimun et al., 2012a; Xu et al., 2013, 2016).

In this study, the excavation and identification of yield-associated QTL would pave the way for cowpea breeding, particularly for marker assisted selection (MAS) in cowpea. The current high-density genetic map is useful for QTL analysis in *V. unguiculata*.

#### Pod length

Pod length, one of the most significant agricultural traits of the yardlong bean (*V. unguiculata* ssp. *sesquipedalis*), is a complex quantitative trait controlled by multiple genes. The “one major QTL + minor QTLs” mode of pod length determination is consistent with an earlier version of the ZZ map (Xu et al., 2011) and the Kongjaimun et al. (2012a) genetic map. The distinct pod trait of the yardlong bean might be the result of oriented domestication for the edible tender pods. Compared with its wild type, the domestication of the yardlong bean has led to an ~8-fold increase in pod length (Pottorff et al., 2014a). The domesticated *V. unguiculata* ssp. *sesquipedalis* parent used here had an average pod length of 78.99 cm, while the domesticated *V. unguiculata* ssp. *unguiculata* had an average pod length of 22.75 cm (Supplementary File 7). The former was almost four times longer than the latter. Four QTL on LGs 4, 6, 9, and 11 were probably involved in pod length variation in *V. unguiculata* (Table 3). Among them, the QTL (*Qcpl-3*) located on LG9 had the greatest effect on pod length. In comparison, regarding the pod-length related traits in *V. unguiculata*, nine QTL (Kongjaimun et al., 2012b) and seven QTL (Kongjaimun et al., 2012a) were revealed in different LGs. The LGs were developed from 226 SSR molecular markers and BC_1_F_1_ and F_2_ populations from the cross between a yardlong bean accession and a wild cowpea accession. Thus, the discrepancy between the pod-length-related QTL numbers and positions in *V. unguiculata* might be attributed to the varied marker types and cross-combination population between the previous investigations and this study. Recently, 72 pod-length-associated SNPs were identified by genome-wide association studies (GWAS), and the gene effects of sugar, gibberellin and nutritional signaling might be involved in the regulation of pod length based on the transcriptomic analysis (Xu et al., 2016). These studies paved the way for the feasibility of cloning pod length genes and marker assisted selection of this trait in cowpea breeding programs.

#### Seed weight (thousand-grain weight)

Two components of seed weight, TGW and grain number per pod (GN), exhibited no obvious difference between the two parents in this study. However, the additive effect of the QTL related to TGW and GN traits in the male parent (*V. unguiculata* ssp. *sesquipedalis*) possibly increased the total seed weight (Table 3, Supplementary File 7).

As to TGW, the average value of TGW trait in F_2:3_ population was 162.61 g, which was closer to the male parent (167.64 g) than the female parent (121.99 g). Four QTL were detected for the TGW trait in cowpea, and that with the largest effect (percentage of explained phenotypic variation 9.85%) was found on LG11 (*Qctgw-4*). This QTL was linked to the interval region of the SNP loci between M6647-M6464 in this study.

Regarding GN, the GN trait was only slightly different between the two parents, although the pod length of the male parent (*V. unguiculata* ssp. *sesquipedalis*) was much longer than that of the female parent (*V. unguiculata* ssp. *unguiculata*). The average grain number per pod of the F_2:3_ population (17 grains) was slightly larger than the female parent (16 grains) but lower than the male parent (20 grains). Two QTL were detected on LGs 5 and 11 for GN. The QTL on LG11 had the largest effect, explaining 6.37% of the phenotypic variation in the F_2:3_ population. As mentioned above, the male parent has a significantly more positive influence than the female parent on the three factors of seed weight. To date, the genomic region associated with seed weight is still unknown, but a domestication-related QTL involved in seed size (*Css-1*) has been observed in cowpea (Lucas et al., 2013b).

#### Plant type

Plant type is a crucial trait in domestication. One aspect of plant type, carpopodium length, showed a slight difference between the two cowpea parents in the present study. The carpopodium length of the F_2:3_ population (mean = 29.58 cm) fluctuated between the female parent *V. unguiculata* ssp. *unguiculata* (32.44 cm) and the male parent *V. unguiculata* ssp. *sesquipedalis* (28.44 cm). Only one QTL was detected in LG10, explaining 2.06% of the phenotypic variation. Unlike the QTL related to seed weight, the allele from the female parent increased the carpopodium length. In addition, the bushy plant architecture is a typical trait of plant type in *V. unguiculata* ssp. *unguiculata*, which was found to correlate with increased pod number per plant in cultivated dry grain cowpea (Bapna et al., 1972). Pod number per plant is another plant type trait studied in cowpea. It was reported that QTL regions of pod length (*Qpl.zaas-3*) overlaps the major QTL for pod number per plant (*Qpn.zaas-3*) (Xu et al., 2013), which may explain the tight correlation between the two traits (Bapna et al., 1972; Aggarwal et al., 1982).

### Comparative analysis between *V. unguiculata* and its closely related legume species

The level of synteny between cowpea and the closely related legume species *V. angularis* was assessed. The Circos plot of the cowpea linkage map vs. *V. angularis* genome domestrated that some syntenic SNP locations were uncovered between the cowpea LGs and the *V. angularis* genome. When comparing the sequences of these QTL regions with reported gene sequences in NCBI databases by BLASTN, we found that two tags (M26270 and M26271) in *Qctgw-2* regions showed a homologous domain with sequences of E3 ubiquitin protein ligase DRIP2-like (LOC106770573) in *V. radiata*. In a previous study, DRIP2 was reported to be a C3HC4 RING–domain protein interacting with the key transcription factor DREB2A and control concentration of DREB2A protein by regulating DREB2A degradation (Qin et al., 2008). In further analysis, it was documented that the DREB2A protein was involved in the seed development in *Arabidopsis thaliana*, because the seed yield of 35S:DREB2A CA-b mutant was lowered to 40% of the wild type of *Arabidopsis thaliana* (Sakuma et al., 2006). Therefore, the region of *Qctgw-2*, which was associated with the trait of thousand grain weight, may have candidate genes that control the seed weight of cowpea.

The SNP type was detected to be different between the two parents in the two tags (M26270 and M26271). In the M26270-SNP tag, the SNP nucleotide is “G” in the female parent, while it is “C” in the male parent. In the M26271-SNP tag, the SNP nucleotide is “C” in the female parent, while it is “A” in the male parent. Considering the phenotypic difference of the seed between the two cowpea parents, the different SNP markers of the parents suggested the feasibility of utilizing marker-assisted selection in breeding programs targeting seed variation in cowpea.

## Author contributions

LP and CC designed the experiments, pipelines of bioinformatics, created the mapping population and wrote the manuscript. ZW, NW, and SG performed the bioinformatics analyses. XY and RG planted the experimental materials and collected the phenotypic data. XY, QX, and YZ performed the Restriction-site associated DNA sequencing (RAD-seq) experiments.

### Conflict of interest statement

The authors declare that the research was conducted in the absence of any commercial or financial relationships that could be construed as a potential conflict of interest.

## Supplementary material

Supplementary Figure 1 Construction of the current mapping population P1: the female parent (“Green pod cowpea”); P2: the male parent (“Xiabao II”).

Supplementary Figure 2 The mapped 17996 SNPs on the 11 LGs of cowpea.

Supplementary Figure 3 The paired SNP marker from the current cowpea linkage map and the Muñoz-Amatriaín et al. (2017) map.

Supplementary Figure 4 LOD value of QTLs of yield-related traits in cowpea **(a)** PL (pod length), **(b)** CL (carpopodium length), **(c)** GN (Grain number per pod), **(d)** TGW (Thousand grain weight).

Supplementary File 1 SNP positions on the 11 LGs of the genetic map in cowpea.

Supplementary File 2 RAD tags of all the 17996 mapped SNPs.

Supplementary File 3 Genome view of synteny between two cowpea linkage groups and common bean genome.

Supplementary File 4 The paired SNPs between two cowpea linkage groups.

Supplementary File 5 Comparison analysis between linkage map of cowpea and adzuki bean genome.

Supplementary File 6 Summary of candidate genes for yield-related QTL in cowpea.

Supplementary File 7 Phenotypic data of all the 170 samples of cowpea.

Supplementary Table 1 Statistics for the identified SNP marker types.

Supplementary Table 2 Summary of comparison analysis between mapped SNPs tags of *V. unguiculata* and *V. angularis* gnome. Chr, chromosome of *Vigna angularis*; LG, linkage group of *Vigna unguiculata*; No. of tags, number of tags.
