# Supplementary figures and images for: A High Density Genetic Map Derived from RAD Sequencing and Its Application in QTL Analysis of Yield-Related Traits in Vigna unguiculata
Source: Front Plant Sci. 2017 Sep 7;8:1544. doi: 10.3389/fpls.2017.01544 (PMC5594218; doi:10.3389/fpls.2017.01544)

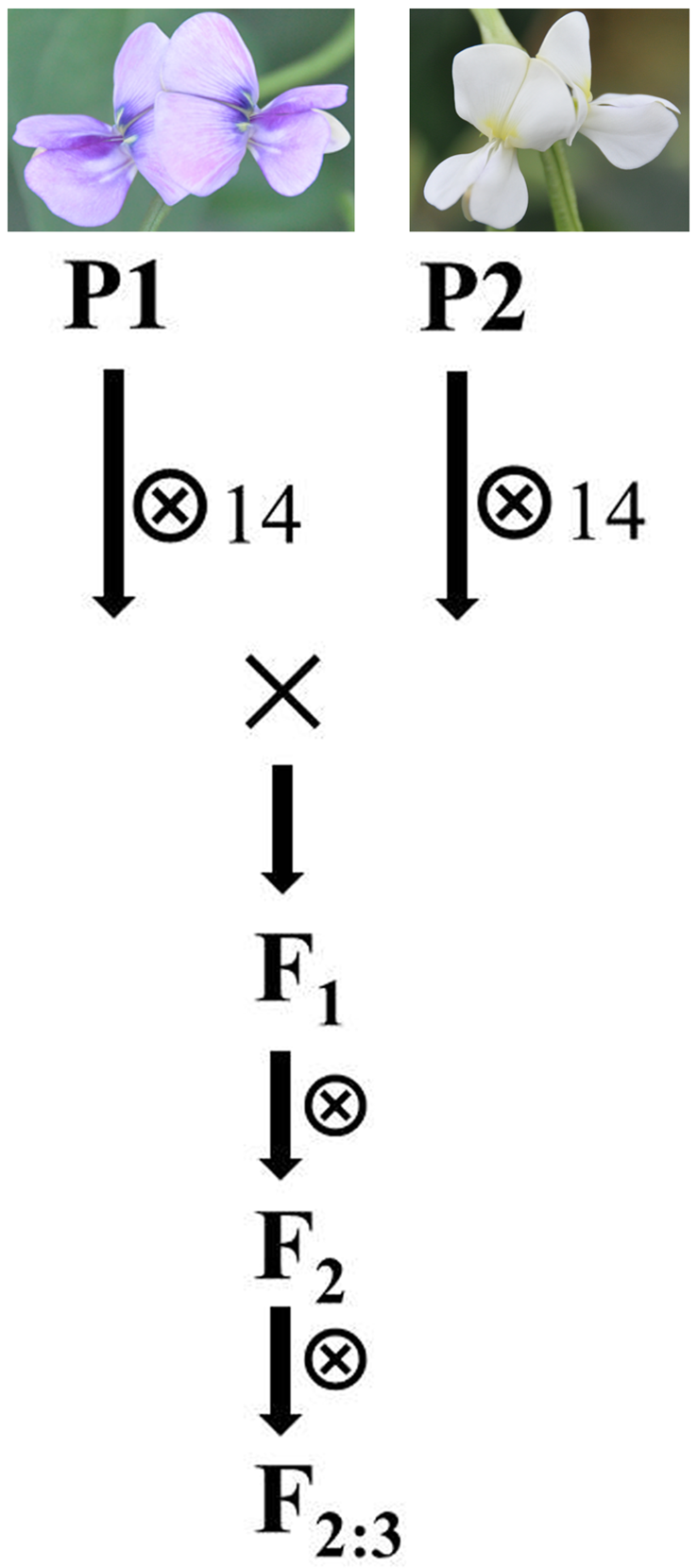

Supplement: Supplementary Figure 1 — Construction of the current mapping population P1: the female parent (“Green pod cowpea”); P2: the male parent (“Xiabao II”). [file Image1.TIF]

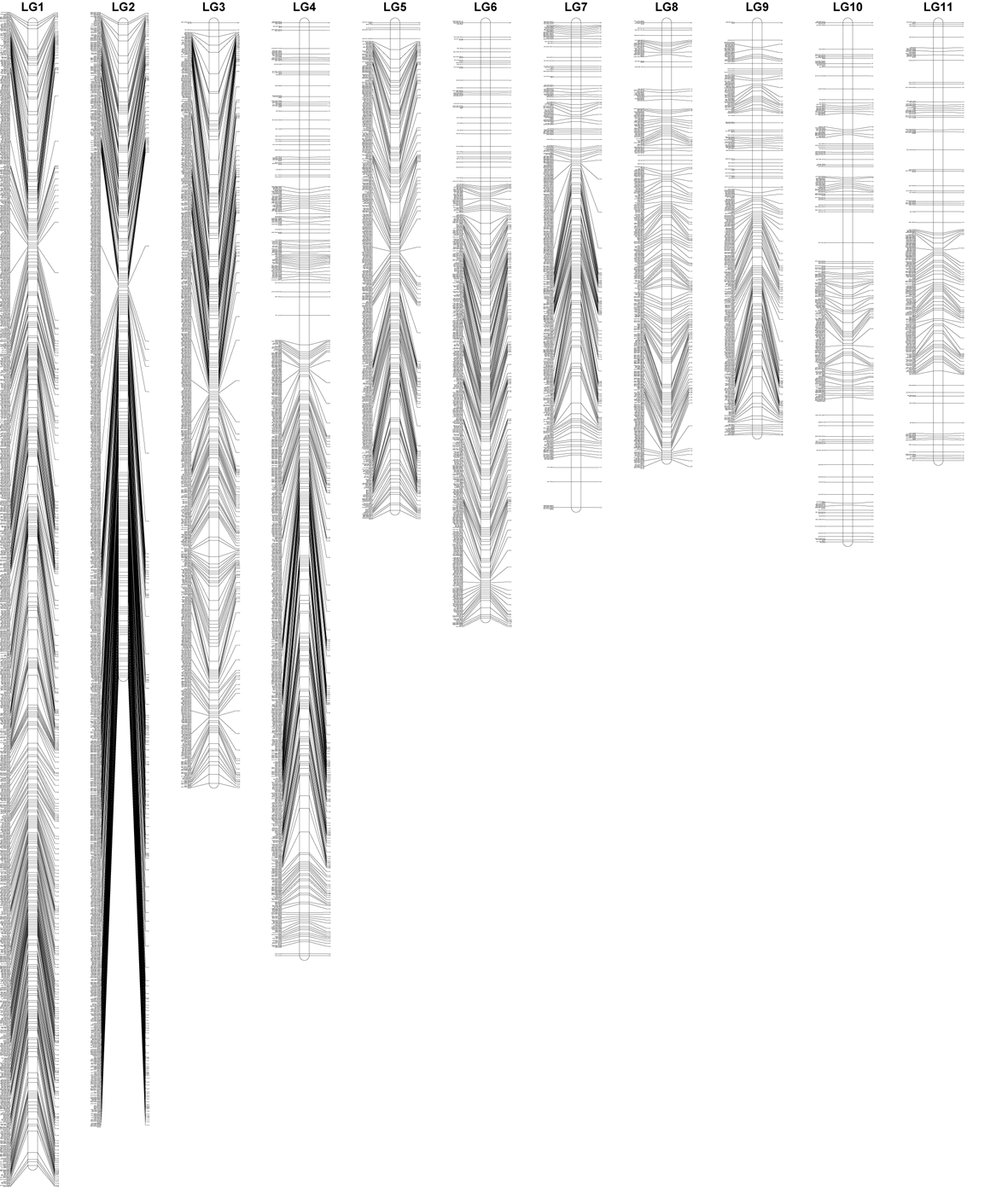

Supplement: Supplementary Figure 2 — The mapped 17996 SNPs on the 11 LGs of cowpea. [file Image2.TIF]

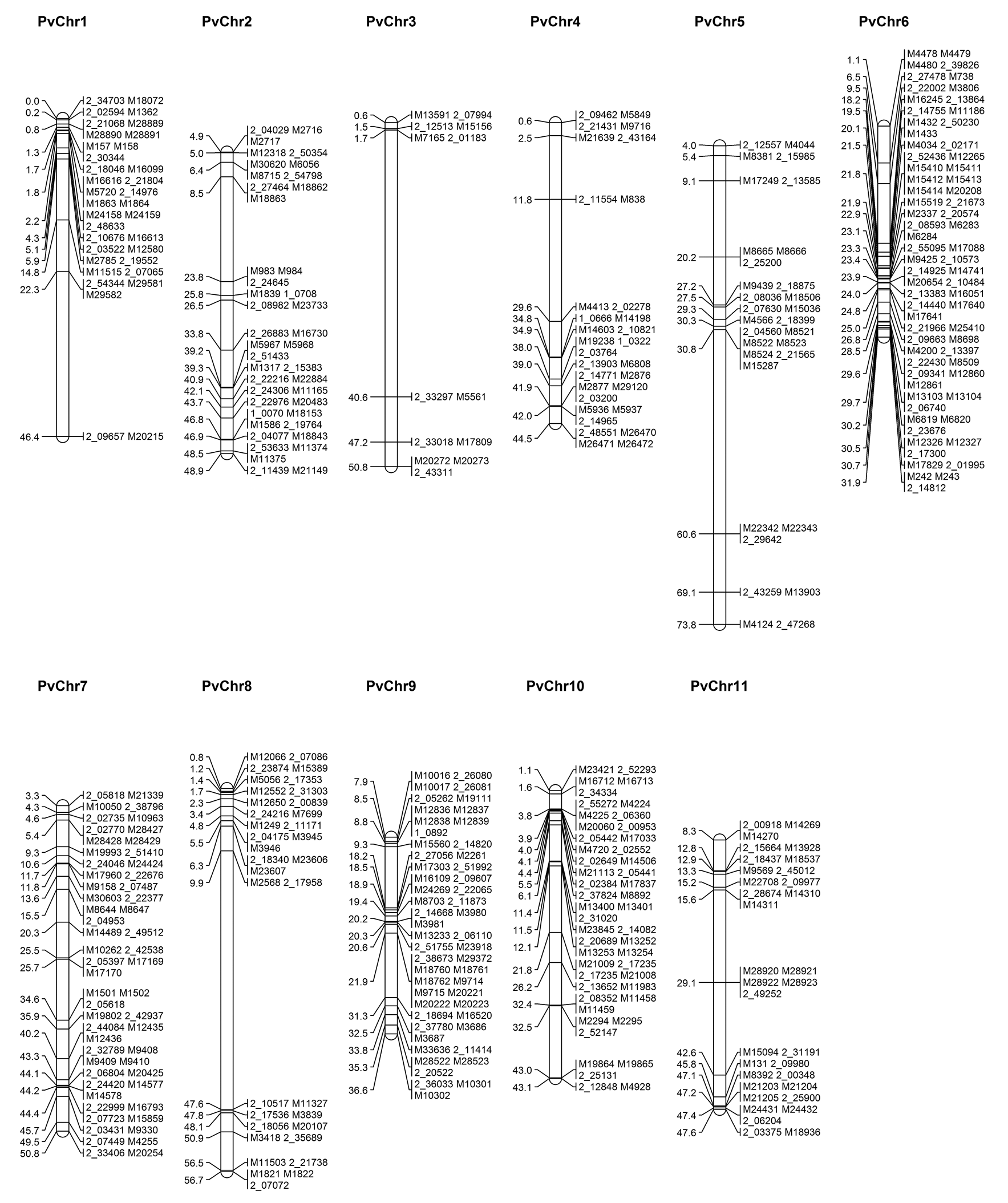

Supplement: Supplementary Figure 3 — The paired SNP marker from the current cowpea linkage map and the Muñoz-Amatriaín et al. (2017) map. [file Image3.TIF]

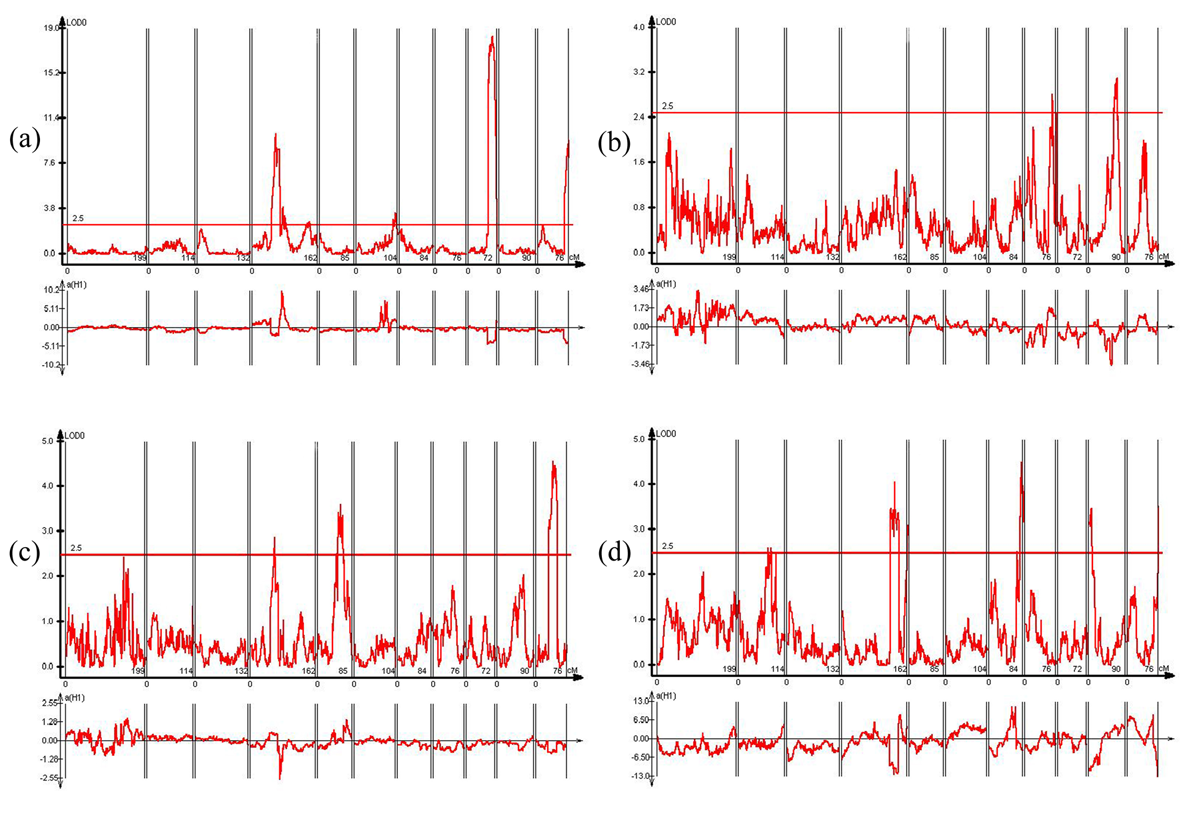

Supplement: Supplementary Figure 4 — LOD value of QTLs of yield-related traits in cowpea (a) PL (pod length), (b) CL (carpopodium length), (c) GN (Grain number per pod), (d) TGW (Thousand grain weight). [file Image4.TIF]
