# Supplementary material for: A High Density Genetic Map Derived from RAD Sequencing and Its Application in QTL Analysis of Yield-Related Traits in Vigna unguiculata
Source: Front Plant Sci. 2017 Sep 7;8:1544. doi: 10.3389/fpls.2017.01544 (PMC5594218; doi:10.3389/fpls.2017.01544)
Supplement: Supplementary Table 1 — Statistics for the identified SNP marker types. [file Table1.doc]

**Supplementary Table 1. Statistics for the identified SNP marker types**

| **SNP type** | **Number** | **Proportion of type (%)** |
| --- | --- | --- |
| C/G | 1122 | 6.2 |
| A/T | 1383 | 7.7 |
| C/A | 1395 | 7.8 |
| G/T | 1396 | 7.8 |
| C/T | 6266 | 34.8 |
| G/A | 6434 | 35.8 |
| total | 17996 | 100.0 |
