# Supplementary material for: A High Density Genetic Map Derived from RAD Sequencing and Its Application in QTL Analysis of Yield-Related Traits in Vigna unguiculata
Source: Front Plant Sci. 2017 Sep 7;8:1544. doi: 10.3389/fpls.2017.01544 (PMC5594218; doi:10.3389/fpls.2017.01544)
Supplement: Supplementary Table 2 — Summary of comparison analysis between mapped SNPs tags of V. unguiculata and V. angularis gnome. Chr, chromosome of Vigna angularis; LG, linkage group of Vigna unguiculata; No. of tags, number of tags. [file Table2.doc]

**Supplementary Table 2. Summary of comparison analysis between mapped SNPs tags of *V. unguiculata* and *V. angularis* gnome.** Chr:chromosome of *Vigna angularis*; LG: linkage group of *Vigna unguiculata* ; No.of tags: number of tags

|  | ***V. angularis*** | | | | | | | | | | | |
| --- | --- | --- | --- | --- | --- | --- | --- | --- | --- | --- | --- | --- |
| ***V. unguiculata*** | **chr1** | **chr2** | **chr3** | **chr4** | **chr5** | **chr6** | **chr7** | **chr8** | **chr9** | **chr10** | **chr11** |  |
| LG1 | 33 | 37 | 25 | 38 | 5 | 30 | 7 | 154 | 6 | 1 | 2 | 338 |
| LG2 | 103 | 139 | 112 | 143 | 42 | 155 | 56 | 78 | 96 | 45 | 72 | 1041 |
| LG3 | 76 | 86 | 75 | 112 | 34 | 111 | 40 | 53 | 73 | 24 | 49 | 733 |
| LG4 | 32 | 35 | 21 | 23 | 6 | 22 | 4 | 6 | 4 | 43 | 134 | 330 |
| LG5 | 146 | 11 | 12 | 19 | 1 | 18 | 3 | 5 | 7 | 3 | 0 | 225 |
| LG6 | 199 | 173 | 179 | 183 | 57 | 259 | 101 | 114 | 131 | 36 | 121 | 1553 |
| LG7 | 11 | 114 | 4 | 18 | 11 | 4 | 2 | 3 | 3 | 1 | 0 | 171 |
| LG8 | 59 | 60 | 311 | 81 | 20 | 80 | 27 | 36 | 45 | 27 | 41 | 787 |
| LG9 | 10 | 13 | 9 | 124 | 1 | 18 | 2 | 7 | 8 | 2 | 8 | 202 |
| LG10 | 3 | 7 | 3 | 3 | 12 | 6 | 4 | 3 | 36 | 1 | 4 | 82 |
| LG11 | 7 | 9 | 5 | 5 | 112 | 3 | 7 | 2 | 1 | 3 | 0 | 154 |
| Total |  |  |  |  |  |  |  |  |  |  |  | 5616 |
